# Injection Drug Use Quality of Life scale (IDUQOL): A validation study

**DOI:** 10.1186/1477-7525-3-43

**Published:** 2005-07-19

**Authors:** Anita M Hubley, Lara B Russell, Anita Palepu

**Affiliations:** 1 Measurement Evaluation and Research Methodology, Dept of ECPS, 2125 Main Mall, The University of British Columbia, Vancouver, BC, Canada; 2 Division of Internal Medicine, Department of Medicine, Faculty of Medicine, University of British Columbia, Vancouver, BC, Canada; 3 Centre for Health Outcome and Evaluation Sciences, St. Paul's Hospital, Vancouver, BC, Canada

**Keywords:** Drug Use, Factor Analysis, Psychometrics, Quality of Life, Reliability, Validity

## Abstract

**Background:**

Existing measures of injection drug users' quality of life have focused primarily on health and health-related factors. Clearly, however, quality of life among injection drug users is impacted by a range of unique cultural, socioeconomic, medical, and geographic factors that must also be considered in any measure. The Injection Drug User Quality of Life (IDUQOL) scale was designed to capture the unique and individual circumstances that determine quality of life among injection drug users. The overall purpose of the present study was to examine the validity of inferences made from the IDUQOL by examining the (a) dimensionality, (b) reliability of scores, (c) criterion-related validity evidence, and (d) both convergent and discriminant validity evidence.

**Methods:**

An exploratory factor analysis using principal axis factoring in SPSS 12.0 was conducted to determine whether the use of a total score on the IDUQOL was advisable. Reliability of scores from the IDUQOL was obtained using internal consistency and one-week test-retest reliability estimates. Criterion-related validity evidence was gathered using variables such as stability of housing, sex trade involvement, high-risk injection behaviours, involvement in treatment programs, emergency treatment or overdose over the previous six months, hospitalization and emergency treatment over the subsequent six month period post data collection. Convergent and discriminant validity evidence was gathered using measures of life satisfaction, self-esteem, and social desirability.

**Results:**

The sample consisted of 241 injection drug users ranging in age from 19 to 61 years. Factor analysis supports the use of a total score. Both internal consistency (alpha = .88) and one-week test-retest reliability (r = .78) for IDUQOL total scores were good. Criterion-related, convergent, and discriminant validity evidence supports the interpretation of IDUQOL total scores as measuring a construct consistent with quality of life.

**Conclusion:**

The findings from this study provide initial evidence to support the use of the IDUQOL total score. The results of the study also suggest the IDUQOL could be further strengthened with additional attention to how some IDUQOL domains are described and satisfaction is measured.

## Background

Existing measures of injection drug users' (IDUs) quality of life (QoL) have focused primarily on health and health-related factors. The Opiate Treatment Index, the only standardized instrument designed specifically for IDUs, is essentially a symptom checklist [1]. The Nottingham Health Profile [2,3] focuses exclusively on health. The MOS surveys (MOS SF-36, MOS SF-20 and MOS-HIV) have been used in IDU populations but because they are constructed to measure the range of health in the general population (with the exception of MOS-HIV), IDUs score very poorly [4,5]. It makes intuitive sense that IDUs have lower physical and psychological health relative to the general population. It is not surprising that IDU scores tend to be clustered at the low end of the distribution and that instruments devised for the general population may not be particularly sensitive to change in the IDU population. For example, some studies found that, in working with HIV patients with a history of injection drug use, some scales measuring the physical aspects of QoL were relatively insensitive to change and that the effects of drug use tended to overshadow the impact of HIV on health [6,7]. Among crack smokers, most SF-36 subscales did not reflect the adverse health effects of crack cocaine use and therefore appeared to have limited applicability with this population [8].

A meta-analysis of existing QoL studies indicated that QoL and health status are distinct constructs that should not be used interchangeably [9]. Of the instruments used with IDUs, only the MOS series examine QoL domains other than health. With the exception of the MOS-HIV (which was adapted for use with HIV patients), the MOS domains were chosen to measure QoL of the general population. Existing QoL tools do not measure the QoL of drug users in a culturally-sensitive fashion [10]. Problems arise with both the item content and methods of administration. These measures clearly do not take into account the full complexity of drug dependence or account for the individual factors that may compromise effective administration. The social context in which drug injectors live is likely a key component of their QoL and most measures do not capture the chronic long-term impact of drug use on diverse domains such as social, psychological, physical and occupational realms [11]. Even instruments such as the MOS-HIV that are devised for HIV-infected individuals are often not applicable to actively using IDUs because the effects of drug use tend to overshadow the impact of HIV [6].

QoL assessment continues to be widely used in clinical trials and observational studies of health and disease to evaluate clinical interventions, treatment side effects, and disease impact over time [12]. It has become evident that population-sensitive approaches that consider the many components of an individual's life that are deemed critical to his/her QoL are needed. Clearly, QoL among IDUs encapsulates a range of unique cultural, socioeconomic, political, medical, and geographic factors that must be considered in measuring QoL. With these considerations in mind, the Injection Drug User Quality of Life (IDUQOL) scale, an instrument that captures the unique and individual circumstances that determine QoL among IDUs, was developed [13]. To be able to use an instrument with confidence, however, one needs to be able to provide evidence of the validity – that is, the meaningfulness, usefulness, and appropriateness – of the inferences to be made from scores obtained on the instrument with a given population and in a given context [14-16]. The overall purpose of the present study was to examine the validity of inferences made from the IDUQOL. Several lines of construct validity evidence were examined: (a) essential unidimensionality supporting use of an IDUQOL total score, (b) internal consistency and test-retest reliability of IDUQOL scores, (c) criterion-related validity evidence, and (d) both convergent and discriminant validity evidence.

## Methods

### Sample

Participants consisted of a sub-sample of individuals participating in the Vancouver Injection Drug User Study (VIDUS), a longitudinal study of the incidence of HIV among IDUs in Vancouver, Canada. The research design and methods of the VIDUS have been previously described [17]. In brief, this open cohort study was initiated in 1996 to clarify the socio-demographic and behavioural determinants of HIV sero-conversion among this group. Eligibility for initial enrolment required current injection drug use (injected at least once within the last month) and evidence of recent injection was required by inspection of needle tracks. Potential participants also were required to reside in the Lower Mainland of British Columbia and provide informed consent. Most participants (82%) were recruited through word of mouth and street outreach programs. The remaining participants were referred by the needle-exchange program (5%), other storefront agencies (10%), and clinics (3%). Participants who have stopped injecting after the baseline visit are still eligible for follow-up. Trained interviewers administer a survey instrument every 6 months. Participants are asked about their demographics, needle sharing, drug using behaviour, sexual behaviours, access to clean needles and syringes, access to health care, service needs, and medical service use (e.g., self-reported visits to primary care/outpatient clinics, Emergency Department, detoxification, methadone maintenance, ambulance use and hospital admissions). Participants were reimbursed $20 CDN for each study visit, at which time referrals were provided for medical care, HIV/AIDS care, available drug and alcohol treatment and counselling as needed. The VIDUS study participants may not be representative of all IDUs because those in the lowest socioeconomic group are overrepresented in this study sample. However, it is this group that is most in need of innovative interventions.

In the present study, a total of 250 individuals were recruited in the order in which they appeared for their regularly scheduled appointment for VIDUS. A subsequent appointment for the quality of life study was scheduled and participants were paid $10 CDN in each session of the present study. Data from nine participants were excluded because of missing data or because they were deemed, at the time of data collection, to be too impaired to focus on the research tasks. The final sample consisted of 241 IDUs ranging in age from 19 to 61 years (M = 39.4, SD = 9.5 years). There were more males (63%) than females (37%) and most participants (85%) had completed high school. There were no significant socio-demographic or drug using behaviour differences between the 250 recruited individuals and the other VIDUS participants.

The first 50 participants were invited to return for a second session within 6–8 days to collect test-retest reliability data. All 50 participants returned for the second session as scheduled. In the test-retest group, 58% were male and 42% were female. These participants ranged in age from 22 to 59 years (M = 41.7, SD = 9.2). Most of the participants (90%) had completed high school.

## Measures

### Injection Drug User Quality of Life Scale (IDUQOL)

The present version of the IDUQOL consists of 21 life domains and builds on the original version first published by Brogly et al. [13]. Many of these domains (e.g., Being Useful, Drugs, Drug Treatment, Harm Reduction and Neighbourhood Safety) are particularly relevant to the physical, social, psychological, occupational, and geographical reality of IDUs' lives. Life domains are each represented on a 5 by 5 inch card, with the name of the domain and a simple representative picture on the front of the card and a description of the domain on the back of the card. Graphic representations were used so that this measure would be more accessible to respondents who do not speak English as a first language or have low literacy skills.

Although the administration of the IDUQOL permitted respondents to subjectively weight the importance of the life domains to his/her quality of life, a review of the literature on importance ratings and weighting [18] as well as an empirical comparison of the utility of weighted versus unweighted scores with the IDUQOL showed that weighting does not improve upon the use of simpler unweighted scores [19]. Thus, unweighted scores are used in the present study, wherein the respondent simply assigned a satisfaction rating for each life domain using a 7-point Likert-type scale ranging from 1 (very dissatisfied) to 7 (very satisfied) and illustrated with seven stylised frowning and smiling faces. Again, visual representation was included as a guide for respondents with limited English or literacy skills. Domain scores were summed and averaged to obtain an overall quality of life score ranging from 1 (very dissatisfied) to 7 (very satisfied).

### Satisfaction with Life Scale (SWLS)

The SWLS is a 5-item global measure of life satisfaction [20]. Scores range from 5 to 35, with higher scores representing greater life satisfaction. This measure was selected because life satisfaction was seen as a related construct to quality of life.

### Rosenberg's Self-Esteem Scale (RSES)

The RSES is a 10-item measure of global self-esteem [21]. Total scores range from 10 to 40, with higher scores representing greater self-esteem. This measure was selected because self-esteem was seen as a related construct to quality of life.

### Marlowe-Crowne Social Desirability Scale Short Form X2 (MC X2)

The MC X2 [22] is a 10-item short form version of the Marlowe-Crowne Social Desirability Scale (MC SDS) [23]. Strahan and Gerbasi reported that it correlates .80 or higher with the MC SDS. The MC X2 provides an estimate of socially desirable responding as a potential source of measurement error. Total scores range from 0 to 10, with higher scores representing higher social desirability in responding. The MC X2 was selected because measures of pervasive characteristics such as social desirability are strongly recommended to assess discriminant validity [24,25].

### Demographic Information

In examining criterion-related validity, the following demographic variables were used to create groups expected to differ in their quality of life: stability of housing, sex trade involvement, high-risk injection behaviours (i.e., lending or borrowing needles, daily use of heroin, cocaine, speed, or crack), involvement in a methadone maintenance program or drug treatment program, reporting hospitalization and emergency department attendance or overdose within the previous six months. Predictive criterion variables included: hospitalization and emergency treatment over the six-month period post-data collection. All variables were measured and coded dichotomously.

### Procedures

Ethics approval for this study was obtained from the University of British Columbia and Providence Health Care Research Ethics Boards. Participants met one-on-one with one of three trained VIDUS staff members for a single session lasting approximately 25–30 minutes. Participants were identified only by their VIDUS study ID code on all research forms utilized for this project. All participants provided informed consent and then completed the study measures in the same order (IDUQOL, MC X2, SWLS, RSES). Demographic and predictive criterion data were obtained from VIDUS using the participants' VIDUS ID codes, a use that was disclosed to participants as part of their informed consent. Retest sessions for the sub-sample of 50 participants followed the same consent process, format, and tasks as the initial session.

## Results

### Essential unidimensionality and use of IDUQOL total score

To be able to use a summed total score on a measure such as the IDUQOL, it is important to demonstrate that the measure shows either strict or essential unidimensionality [26,27]. Strict unidimensionality denotes the presence of a single common factor whereas essential unidimensionality indicates the presence of a reasonably dominant common factor along with secondary minor dimensions [28,29].

**Figure 1:**
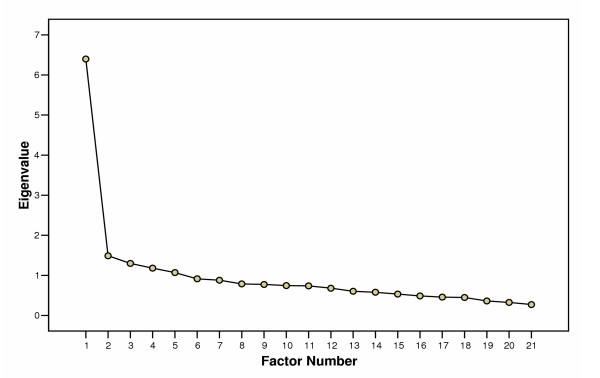
Scree Plot Showing Eigenvalues for Each Possible Factor of the IDUQOL.

An exploratory factor analysis using principal axis factoring in SPSS 12.0 was conducted on the 21 items of the IDUQOL to determine whether essential unidimensionality was present and supported the use of the IDUQOL total score. According to Gorsuch's guideline of 5 to 10 cases per item [30], the sample size for the present study (n = 241) was considered adequate for factor analysis of the 21-item IDUQOL. The data met the criteria for Bartlett's Test of Sphericity, χ^2^(210) = 1488.02, p < 0.001 and the Kaiser-Meyer-Olkin criteria for sampling adequacy, KMO = .88 [31]. The first factor had an eigenvalue of 6.40 and explained 30.5% of the variance in participants' responses. The ratio of the first to the second eigenvalue was 4.3 which exceeds the strict criterion of a ratio greater than 4.0 for evidence of unidimensionality [30,32,33]. These results, in addition to a visual examination of the scree plot (see Figure 1) indicated an essentially unidimensional factor structure for the IDUQOL, which supported the use of a total score [32-35]. Factor loadings ranged from .31 to .71 for all IDUQOL items on a single factor.

### Mean performance and reliability

Table 1 displays the inter-item correlation matrix for the IDUQOL. The mean inter-item correlation was .26, which Clark and Watson [36] describe as acceptable. Table 2 shows the mean performance and internal consistency of scores obtained by the sample on the IDUQOL, SWLS, RSES, and MC X2. Given the focus in the present study on the IDUQOL, gender differences on scores from this measure were also examined. No statistically significant differences in performance on the IDUQOL were found between men (M = 4.25, SD = 0.96) and women (M = 4.10, SD = 1.02), t (239) = 1.14, p = .20, and the effect size (d = 0.15) is considered small according to Cohen [37].

**Table 1:** Inter-item Correlations on the IDUQOL

Items	BU	DR	DT	ED	FA	FG	FR	HR	HE	HC	HO	IN	LA	MO	NS	PA	RC	SX	SP	TR
BU	1.00																			
DR	0.27	1.00																		
DT	0.14	0.37	1.00																	
ED	0.36	0.13	0.14	1.00																
FA	0.35	0.12	0.15	0.13	1.00															
FG	0.54	0.43	0.26	0.25	0.34	1.00														
FR	0.31	0.22	0.20	0.28	0.35	0.52	1.00													
HR	0.21	0.21	0.22	0.17	0.10	0.15	0.15	1.00												
HE	0.29	0.38	0.25	0.13	0.25	0.43	0.29	0.13	1.00											
HC	0.17	0.20	0.40	0.19	0.23	0.29	0.27	0.18	0.35	1.00										
HO	0.23	0.30	0.21	0.16	0.30	0.33	0.40	0.10	0.40	0.37	1.00									
IN	0.39	0.34	0.31	0.25	0.28	0.48	0.37	0.22	0.31	0.40	0.31	1.00								
LA	0.45	0.35	0.23	0.28	0.22	0.38	0.37	0.22	0.16	0.30	0.31	0.31	1.00							
MO	0.35	0.26	0.16	0.30	0.20	0.34	0.33	0.14	0.28	0.31	0.28	0.31	0.41	1.00						
NS	0.31	0.18	0.24	0.19	0.22	0.32	0.42	0.26	0.29	0.36	0.36	0.37	0.34	0.34	1.00					
PA	0.22	0.14	0.18	0.12	0.26	0.29	0.27	0.04	0.18	0.14	0.26	0.24	0.15	0.11	0.21	1.00				
RC	0.18	0.09	0.18	0.24	0.17	0.20	0.29	0.25	0.12	0.27	0.13	0.26	0.25	0.26	0.24	0.11	1.00			
SX	0.24	0.18	0.12	0.14	0.36	0.29	0.33	0.06	0.26	0.19	0.25	0.24	0.24	0.23	0.22	0.60	0.20	1.00		
SP	0.27	0.25	0.28	0.25	0.29	0.45	0.37	0.17	0.21	0.16	0.18	0.29	0.30	0.24	0.17	0.18	0.11	0.20	1.00	
TR	0.29	0.18	0.19	0.34	0.19	0.27	0.34	0.14	0.21	0.22	0.34	0.30	0.34	0.36	0.25	0.10	0.28	0.19	0.21	1.00
TO	0.39	0.29	0.29	0.24	0.25	0.46	0.50	0.13	0.39	0.37	0.34	0.43	0.35	0.31	0.40	0.17	0.24	0.18	0.31	0.30

Items: BU = Being Useful; DR = Drugs; DT = Drug Treatment; ED = Education; FA = Family; FG = Feeling Good; FR = Friends; HR = Harm Reduction; HE = Health; HC = Health Care; HO = Housing; IN = Independence; LA = Leisure Activities; MO = Money; NS = Neighbourhood Safety; PA = Partner(s); RC = Resources in the Community; SX = Sex; SP = Spirituality; TR = Transportation; TO = Treatment by Others.

**Table 2:** Mean Performance and Reliability on the IDUQOL, MC X2, SWLS, and RSES

	Possible Score Range	Actual Score Range	Mean (Standard Deviation)	Internal Consistency
IDUQOL	0 – 7	1.9 – 6.7	4.19 (0.98)	.88
SWLS	5 – 35	5 – 32	14.44 (7.17)	.85
RSES	10 – 40	11 – 40	27.39 (4.96)	.82
MC X2	0 – 10	0 – 10	4.53 (2.10)	.62

IDUQOL = Injection Drug User Quality of Life Scale, SWLS = Satisfaction with Life Scale, RSES = Rosenberg Self Esteem Scale, MC X2 = Marlowe-Crowne Social Desirability Short Form X2. Internal consistency reliability estimates were obtained using Cronbach's coefficient alpha.

In addition to an internal consistency reliability estimate, the one-week test-retest reliability estimate for the IDUQOL scores was also computed. Based on the sub-sample of 50 participants who completed the measure twice, the test-retest reliability estimate was .78, with correlations for each domain across the two sessions ranging from .32 to .67. Table 3 shows the test-retest correlations for all domains.

**Table 3:** One Week Test-Retest Reliability Estimates for the IDUQOL Domain and Total Scores

IDUQOL Domain	Reliability Estimate
Being Useful	.60**
Drugs	.59**
Drug Treatment	.32*
Education	.44**
Family	.43**
Feeling Good	.67**
Friends	.66**
Harm Reduction	.47**
Health	.44**
Health Care	.44**
Housing	.63**
Independence	.57**
Leisure Activities	.62**
Money	.55**
Neighbourhood Safety	.65**
Partner(s)	.64**
Community Resources	.34*
Sex	.52**
Spirituality	.57**
Transportation	.59**
Treatment by Others	.49**

IDUQOL Total Score	.78**

* p <.05, ** p <.01; N = 50

### Criterion-related validity evidence

Table 4 shows the correlations of the IDUQOL total scores with the dichotomously scored criterion variables. Of the statistically significant correlations, all were in the expected direction. That is, lower IDUQOL scores were related to unstable housing, sex trade involvement, borrowing and lending needles, daily use of heroin and speed, and overdose in the past six months. The IDUQOL scores did not correlate significantly with daily use of cocaine or crack, methadone or drug treatment, emergency treatment, or hospitalization within the six months prior to, or following, the initial test session.

**Table 4:** Correlations of IDUQOL Total Scores with Criterion Measures

Criterion Variable	IDUQOL Total Score
Housing (stable = 0/ unstable = 1)	-.16*
Engaged in sex trade (no = 0/yes = 1)	-.17**
Currently borrowing needles (no = 0/yes = 1)	-.19**
Currently lending needles (no = 0/yes = 1)	-.25**
At least once daily use of heroin (no = 0/yes = 1)	-.26**
At least once daily use of cocaine (no = 0/yes = 1)	-.11
At least once daily use of speed (no = 0/yes = 1)	-.14*
At least once daily use of crack (no = 0/yes = 1)	-.12
Currently on methadone treatment (no = 0/yes = 1)	.07
Drug treatment program in last 6 months (no = 0/yes = 1)	.01
Overdose in last 6 months (no = 0/yes = 1)	-.14*
Visited ER in last 6 months (no = 0/yes = 1)	-.06
Hospitalized in last 6 months (no = 0/yes = 1)	-.06
Visited ER in subsequent 6 months (no = 0/yes = 1)	-.05
Hospitalized in subsequent 6 months (no = 0/yes = 1)	.01

*p < .05, **p < .01; N = 241

IDUQOL scores are based on a wide range of domains that encompass social, physical and emotional realms, and therefore, as a total score, might not correlate significantly with specific criterion variables. To explore this possibility, analyses were carried out at the domain level, matching available criterion variables with relevant IDUQOL domains. For example, the criterion variables of engaged in sex trade and Rosenberg Self-Esteem Scale scores were correlated with the Feeling Good about Yourself IDUQOL domain score. Table 5 shows the correlations of selected IDUQOL domain scores and corresponding criterion variables.

**Table 5:** Correlations of Selected IDUQOL Domain Scores with Selected Criterion Variables

**Criterion Variable**	**IDUQOL Domains**
	**Drugs**
At least once daily use of heroin (no = 0/yes = 1)	-.13
At least once daily use of cocaine (no = 0/yes = 1)	-.07
At least once daily use of speed (no = 0/yes = 1)	-.12
At least once daily use of crack (no = 0/yes = 1)	-.07
	**Drug Treatment**
Currently on methadone treatment (no = 0/yes = 1)	.19**
Drug treatment program in last 6 months (no = 0/yes = 1)	.21**
	**Feeling Good About Yourself**
Rosenberg Self Esteem Scale	.58**
Engaged in sex trade (no = 0/yes = 1)	-.20**
	**Health**
Currently on methadone treatment (no = 0/yes = 1)	.06
Drug treatment program in last 6 months (no = 0/yes = 1)	.01
Visited ER in last 6 months (no = 0/yes = 1)	-.14*
Hospitalized in last 6 months (no = 0/yes = 1)	-.16*
	**Health Care**
Visited ER in last 6 months (no = 0/yes = 1)	.03
Hospitalized in last 6 months (no = 0/yes = 1)	.003
	**Housing**
Housing (stable = 0/ unstable = 1)	-.30**
	**How Others Treat You**
Engaged in sex trade (no = 0/yes = 1)	-.15*

*p < .05, **p < .01; N = 241

### Convergent and discriminant validity evidence

Table 6 shows the correlations of IDUQOL total scores with the SWLS, RSES, and MC X2. The convergent measures (SWLS, RSES) showed moderately high correlations with the IDUQOL as would be expected between constructs that are related but not the same. The correlation between the IDUQOL and the discriminant measure (MC X2) was in the low to moderate range and thus acceptable [38]. As expected, the convergent measures were both more highly correlated with the IDUQOL total score than was the discriminant measure. Correlations were also conducted between the MC X2 and both the SWLS (r = .35) and RSES (r = .41). Because the relationship between the IDUQOL and the convergent measures could be due to the common influence of social desirability bias, partial correlations between the IDUQOL total scores and the SWLS and RSES, controlling for MC X2 scores, were conducted. These are reported in Table 6.

**Table 6:** Correlations and Partial Correlations of IDUQOL Total Scores With Convergent and Discriminant Measures

	Correlations with IDUQOL Total Score^a^	Partial Correlations with IDUQOL Total Score^b^
**Convergent Measures**		
Satisfaction With Life Scale	.59**	.54**
Rosenberg Self Esteem Scale	.54**	.47**
**Discriminant Measure**		
Marlowe-Crowne Social Desirability Scale (MC X2)	.35**	

**p < .01; ^a^N = 241; ^b^Partial correlations control for MC X2 scores, N = 238

## Discussion

The IDUQOL was developed to be a more appropriate and sensitive measure of quality of life for IDUs within their unique context of social, psychological, physical, occupational, and geographical factors. This study was designed to examine the construct validity of inferences made from the IDUQOL by exploring the factor structure, reliability, criterion-related validity evidence, and convergent and discriminant validity evidence. The exploratory factor analysis using principal axis factoring indicates the presence of essential unidimensionality, which, in turn, supports the use of a total score for the IDUQOL. Internal consistency and one week test-retest reliability estimates for the IDUQOL total score were satisfactory.

Criterion-related validity evidence for inferences made from IDUQOL total scores is weak. That is, although lower IDUQOL total scores were statistically significantly related to unstable housing, involvement in the sex trade, borrowing and lending needles, daily use of heroin and speed, and overdose in the previous six months, the correlations were low (r = -.14 to -.26). Moreover, IDUQOL total scores did not correlate significantly with daily use of cocaine or crack, methadone or drug treatment, emergency treatment within the previous six months, or hospitalization within the following six months (r = -.12 to .07). These results may not be too surprising, however, given that the IDUQOL measures numerous life domains.

When specific criterion variables were correlated with individual IDUQOL domains, some showed considerably stronger correlations (e.g., Rosenberg Self-Esteem Scale correlated .58 with the Feeling Good about Yourself domain; instability of housing correlated -.30 with the Housing domain; drug treatment program and methadone treatment correlated .21 and .19, respectively, with the Drug Treatment domain). The fact that the correlations between other criterion variables and specific domains did not change appreciably or even declined (e.g., daily use of specific drugs with the Drugs domain) suggests that there may be some lack of consistency in how participants interpreted the IDUQOL domains. For example, when rating their level of satisfaction with the Drugs domain, it is not clear whether individual participants may have indicated dissatisfaction because of a lack of availability of drugs or because of the impact of drugs in their lives. As a result, this lack of clarity may produce low or near-zero correlations between criterion variables and some IDUQOL domain ratings. In other cases, correlations may be low because of low variability (e.g., mortality) or reduced information (e.g., dichotomous (yes/no) rather than continuous (actual number) measurement of overdoses in previous six months) in the criterion variables. In future criterion-related validity research involving the IDUQOL, some criterion variables may need to be measured differently to improve the variability in scores.

These results suggest that improvements can be made to how (a) some IDUQOL domains are described, and (b) satisfaction is measured that would strengthen the utility of this measure. Individual qualitative interviews with IDUs to explore how individuals are interpreting the IDUQOL domains and assigning satisfaction ratings would provide important guidance on the types of modifications to be made. More importantly, further consideration needs to be given to how the IDUQOL can be used effectively as an outcome measure in intervention studies in which programs addressing specific aspects of quality of life (e.g., housing, health) are evaluated.

Convergent and discriminant validity evidence for the IDUQOL was strong. Convergent measures (SWLS, RSES) correlated more highly with the IDUQOL total scores than was the discriminant measure (MC X2). The moderate (r = .54 to .59) correlations between the IDUQOL total scores and the measures of related, but not identical, constructs of life satisfaction and self-esteem are to be expected. The finding of a significant but low moderate correlation of .35 between the IDUQOL total scores and the MC X2 provides evidence to support discriminant validity but also suggests social desirability plays some role in participants' responses. A similar relationship was found between the MC X2 and both the SWLS and RSES. Because the relationship between the IDUQOL and the convergent measures could be due to the common influence of social desirability bias, partial correlations between the IDUQOL total scores and the SWLS and RSES, controlling for MC X2 scores, were examined. The results showed that, although the magnitude of these correlations declined slightly, the relationships between the IDUQOL and both the SWLS and RSES were not due to social desirability bias.

## Conclusion

The findings from this study provide preliminary evidence to support the meaningfulness, usefulness, and appropriateness of inferences made from IDUQOL total scores. Factor analysis supports the use of a total score. Both internal consistency (Cronbach alpha = .88) and one-week test-retest reliability (r = .78) for IDUQOL total scores are good. Convergent and discriminant validity evidence supports the interpretation of IDUQOL total scores as measuring a construct consistent with quality of life and yet distinctive from life satisfaction, self-esteem, and social desirability bias. The criterion-related validity evidence is weak, but also suggests that the utility of the IDUQOL could be further improved with greater attention to how some IDUQOL domains are described, how satisfaction is measured, and how the IDUQOL and its domains may be applied in both the development and evaluation of various interventions (e.g., drug treatment programs, health and clinical interventions, and social programs).

## List of abbreviations

IDUQOL injection drug user quality of life scale

QoL quality of life

IDUs injection drug users

VIDUS Vancouver Injection Drug User Study

HIV Human immunodeficiency virus

SWLS Satisfaction with Life Scale

RSES Rosenberg's Self-Esteem Scale

MC SDS Marlowe-Crowne Social Desirability Scale

MC X2 Marlowe-Crowne Social Desirability Scale Short Form X2

## Authors' contributions

AH obtained funding, designed the study, directed the statistical analyses, prepared the initial draft of the manuscript and conducted revisions. LR assisted in preparing the data, performed statistical analyses and assisted with revisions. AP conceived of the study, obtained funding, coordinated data collection, and conducted revisions of the manuscript. All authors read and approved the final manuscript.
